# Supplementary material for: Trends in suicide rates by race and ethnicity among members of the United States Army
Source: PLoS One. 2023 Jan 17;18(1):e0280217. doi: 10.1371/journal.pone.0280217 (PMC9844903; doi:10.1371/journal.pone.0280217)
Supplement: S1 Table — (DOCX) [file pone.0280217.s001.docx]

**Supplemental 1 Table. Sample Characteristics by First Deployers and 2+ Deployers within Component**

|  | **Active Duty** | | **National Guard** | | **Reserve** | |
| --- | --- | --- | --- | --- | --- | --- |
|  | First Deployers  (N=386,283) | 2+ Deployers  (N=187,248) | First Deployers  (N=152,539) | 2+ Deployers  (N=53,793) | First Deployers  (N=59,513) | 2+ Deployers  (N=21,554) |
| **Age Category at**  **End of Index Deployment** |  |  |  |  |  |  |
| 18-24 | 202,884  (52.5%) | 32,233  (17.2%) | 60,110  (39.4%) | 4541  (8.4%) | 19,313  (32.5%) | 1467  (6.8%) |
| 25-29 | 93,762  (24.3%) | 59,156  (31.6%) | 32,296  (21.2%) | 13,718  (25.5%) | 12,626  (21.2%) | 5717  (26.5%) |
| 30-34 | 39,752  (10.3%) | 39,854  (21.3%) | 17,779  (11.7%) | 9907  (18.4%) | 6775  (11.4%) | 3518  (16.3%) |
| 35-39 | 27,386  (7.1%) | 31,056  (16.6%) | 15,489  (10.2%) | 8951  (16.6%) | 6185  (10.4%) | 2935  (13.6%) |
| 40+ | 22,499  (5.8%) | 24,949  (13.3%) | 26,865  (17.6%) | 16,676  (31.0%) | 14,614  (24.6%) | 7917  (36.7%) |
| **Gender** |  |  |  |  |  |  |
| Male | 340,051  (88.0%) | 172,036  (91.9%) | 135,523  (88.8%) | 50,855  (94.5%) | 49,047  (82.4%) | 18,977  (88.0%) |
| Female | 46,232  (12.0%) | 15,212  (8.1%) | 17,016  (11.2%) | 2938  (5.5%) | 10,466  (17.6%) | 2577  (12.0%) |
| **Race/Ethnicity** |  |  |  |  |  |  |
| American Indian/Alaskan Native | 3502  (0.9%) | 1684  (0.9%) | 1474  (1.0%) | 515  (1.0%) | 553  (0.9%) | 190  (0.9%) |
| Asian or Pacific Islander | 39,209  (10.2%) | 19,678  (10.5%) | 4136  (2.7%) | 1741  (3.2%) | 2912  (4.9%) | 1023  (4.8%) |
| Black non-Hispanic | 65,587  (17.0%) | 37,505  (20.0%) | 20,013  (13.1%) | 6147  (11.4%) | 10,624  (17.9%) | 3474  (16.1%) |
| White non-Hispanic | 229,802  (59.5%) | 103,602  (55.3%) | 113,303  (74.3%) | 41,035  (76.3%) | 37,629  (63.2%) | 14,063  (65.3%) |
| Hispanic | 44,229  (11.5%) | 21,246  (11.4%) | 12,133  (8.0%) | 3699  (6.9%) | 7417  (12.5%) | 2641  (12.3%) |
| Other | 3042  (0.8%) | 3161  (1.7%) | 901  (0.6%) | 470  (0.9%) | 162  (0.3%) | 102  (0.5%) |
| Unknown/Missing | 912  (0.2%) | 372  (0.2%) | 579  (0.4%) | 186  (0.4%) | 216  (0.4%) | 61  (0.3%) |
| **Rank Group** |  |  |  |  |  |  |
| Junior Enlisted | 255,810  (66.2%) | 35,464  (18.9%) | 85,006  (55.7%) | 9489  (17.6%) | 25,281  (42.5%) | 2413  (11.2%) |
| Senior Enlisted/Warrant Officer | 83,653  (21.7%) | 126,361 (67.5%) | 53,478 (35.1%) | 37,882 (70.4%) | 23,784 (40.0%) | 14,047 (65.2%) |
| Junior Officer | 35,916  (9.3%) | 13,508  (7.2%) | 10,075  (6.6%) | 3521  (6.6%) | 5453  (9.2%) | 1727  (8.0%) |
| Senior Officer | 10,902  (2.8%) | 11,915  (6.4%) | 3979  (2.6%) | 2900  (5.4%) | 4994  (8.4%) | 3367  (15.6%) |
| Missing | 2 (0%) | 0 | 1 (0%) | 1 (0%) | 1 (0%) | 0 |
| **Fiscal Year of Return from index deployment** |  |  |  |  |  |  |
| FY2008-FY2009 | 128,896  (33.4%) | 94,458  (50.5%) | 51,396  (33.7%) | 16,412  (30.5%) | 18,847  (31.7%) | 6411  (29.7%) |
| FY2010-FY2011 | 131,143  (34.0%) | 78,377  (41.9%) | 56,736  (37.2%) | 27,241  (50.6%) | 21,653  (36.4%) | 10,951  (50.8%) |
| FY2012-FY2014 | 126,244  (32.7%) | 14,413  (7.7%) | 44,407  (29.1%) | 10,140  (18.9%) | 19,013  (32.0%) | 4192  (19.5%) |
