# Supplementary material for: Trends in suicide rates by race and ethnicity among members of the United States Army
Source: PLoS One. 2023 Jan 17;18(1):e0280217. doi: 10.1371/journal.pone.0280217 (PMC9844903; doi:10.1371/journal.pone.0280217)
Supplement: S2 Table — (DOCX) [file pone.0280217.s002.docx]

**Supplemental 2 Table. Hazard Rates by Years Since End of Index Deployment**

|  | **Years Since End of Index Deployment** | | | | | | | | |
| --- | --- | --- | --- | --- | --- | --- | --- | --- | --- |
| **Race /Ethnicity** | 0-1 year | 1-2 years | 2-3 years | 3-4 years | 4-5 years | 5-6 years | 6-7 years | 7-8 years | 8-11 years |
| Asian or Pacific Islander | 34.96  (21.0, (48.9) | 45.21  (29.3, 61.1) | 46.74  (30.5, 62.9) | 42.41  (27.0, 57.9) | 38.51  (23.7, 53.3) | 32.29  (18.5, 46.1) | 37.40  (22.1, 52.7) | 42.65  (25.6, 59.7) | 46.30  (31.6, 61.0) |
| Black non-Hispanic | 20.24  (12.9, 27.6) | 22.36  (14.6, 30.1) | 23.79  (15.8, 31.8) | 29.42  (20.5, 38.3) | 25.02  (16.7, 33.3) | 23.00  (14.8, 31.2) | 23.02  (14.3, 31.7) | 20.96  (12.0, 29.9) | 18.94  (11.8, 26.1) |
| White non-Hispanic | 38.59  (33.3, 43.8) | 40.87  (35.5, 46.3) | 39.81  (34.5, 45.1) | 40.24  (34.9, 45.6) | 44.66  (38.9, 50.4) | 45.60  (39.7, 51.5) | 43.16  (37.1, 49.2) | 42.81  (36.2, 49.4) | 41.47  (36.0, 47.0) |
| Hispanic | 23.00  (13.2, 32.8) | 21.93  (12.3, 31.5) | 24.14  (14.1, 34.2) | 18.67  (9.80, 27.5) | 26.97  (16.2, 37.8) | 32.45  (20.2, 44.7) | 45.22  (30.0, 60.4) | 32.87  (18.8, 46.9) | 24.15  (14.1, 34.2) |

*Note.* Hazard rates are estimated at the midpoint of each interval. Green shading indicates rates<20 per 100,000 alive at the beginning of the

interval, yellow indicates rates ≥20 to <30 per 100,000, orange indicates rates ≥30 to <40 per 100,000, and red indicates rates >40 per 100,000.
